# ﻿Four new species of the spider genus *Liphistius* (Araneae, Mesothelae, Liphistiidae, Liphistiinae) from Myanmar

**DOI:** 10.3897/zookeys.1210.123986

**Published:** 2024-08-16

**Authors:** Xin Xu, Yi Zhan, Khin Pyae Pyae Aung, Fengxiang Liu, Long Yu, Daiqin Li

**Affiliations:** 1 College of Life Sciences, Hunan Normal University, 36 Lushan Road, Changsha 410081, Hunan Province, China Hunan Normal University Changsha China; 2 State Key Laboratory of Biocatalysis and Enzyme Engineering, and Centre for Behavioural Ecology and Evolution (CBEE), School of Life Sciences, Hubei University, 368 Youyi Road, Wuhan 430062, Hubei Province, China Hubei University Wuhan China; 3 Department of Zoology, University of Yangon, Kamayut Township, Pyay Road, Yangon, 11041, Myanmar University of Yangon Yangon Myanmar; 4 Department of Biology, Taungoo Education Degree College, Taungoo, 08101, Myanmar Taungoo Education Degree College Taungoo Myanmar; 5 Department of Biological Sciences, National University of Singapore, 14 Science Drive 4, Singapore 117543 National University of Singapore Singapore Singapore

**Keywords:** description, Liphistiomorphae, morphology, southeast Asia, taxonomy

## Abstract

Four new species of *Liphistius* belonging to the *birmanicus* species group are described from Myanmar based on both sexes: *L.kalaw* Zhan & Xu, **sp. nov.** (♂♀), *L.kanpetlet* Zhan & Xu, **sp. nov.** (♂♀), *L.nawngau* Zhan & Xu, **sp. nov.** (♂♀) and *L.rostratus* Zhan & Xu, **sp. nov.** (♂♀). Currently, Myanmar stands as the westernmost country where *Liphistius* is distributed, with the new species *L.kanpetlet***sp. nov.** being found in the westernmost region of Myanmar.

## ﻿Introduction

Currently, the spider family Liphistiidae includes eight genera divided into two subfamilies, the monotypic Liphistiinae Thorell, 1869 and Heptathelinae Kishida, 1923. *Liphistius* Schiödte, 1849, the sole genus of Liphistiinae, encompasses 77 valid species. Among these, six are known solely from females, while the remaining 71 species are described based on both sexes (WSC 2024). The geographical distribution of *Liphistius* spans from its northernmost point in China (Yunnan Province) to its southernmost in Indonesia (Sumatra), while its range extends from the easternmost areas of Laos, Thailand, and Peninsular Malaysia to the westernmost regions of Myanmar (WSC 2024). Until now, several regional revisions of *Liphistius* have been conducted, including those focused on Peninsular Malaysia, Myanmar, and Thailand (for more details, refer to Schwendinger 2017; Sivayyapram et al. 2017; Schwendinger et al. 2019, 2022).

**Figure 1.**
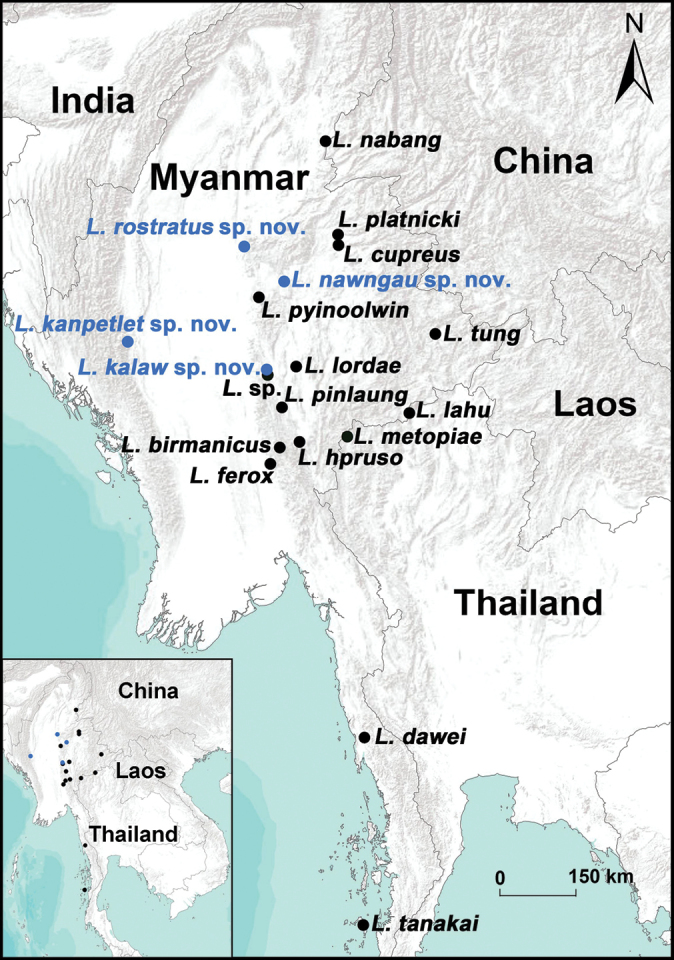
Map showing the collecting localities of the *Liphistius* species in Myanmar, China (*L.nabang*), and Thailand (*L.lahu*, *L.metopiae*). Blue circles refer to four new species described in this study, black circles indicate known and one putative species.

Schwendinger (1990, 2017), Sivayyapram et al. 2017, and Schwendinger et al. 2022 split *Liphistius* into seven species groups based on the morphology of copulatory organs of both sexes: *batuensis*-group (e.g., *L.batuensis* Abraham, 1923), *birmanicus*-group (e.g., *L.birmanicus* Thorell, 1897), *bristowei*-group (e.g., *L.bristowei* Platnick & Sedgwick, 1984), *linang*-group (e.g., *L.linang* Schwendinger, 2017), *malayanus*-group (e.g., *L.malayanus* Abraham, 1923), *tioman*-group (e.g., *L.tioman* Platnick & Sedgwick, 1984), and *trang*-group (e.g., *L.trang* Platnick & Sedgwick, 1984). Currently, Myanmar hosts 11 known *Liphistius* species, including one species known only from females (Fig. 1; WSC 2024). These species belong to three groups: the *birmanicus*-group, the *bristowei*-group, and the *trang*-group (Schwendinger 1990; Schwendinger et al. 2022; Sivayyapram et al. 2024).

**Figure 2.**
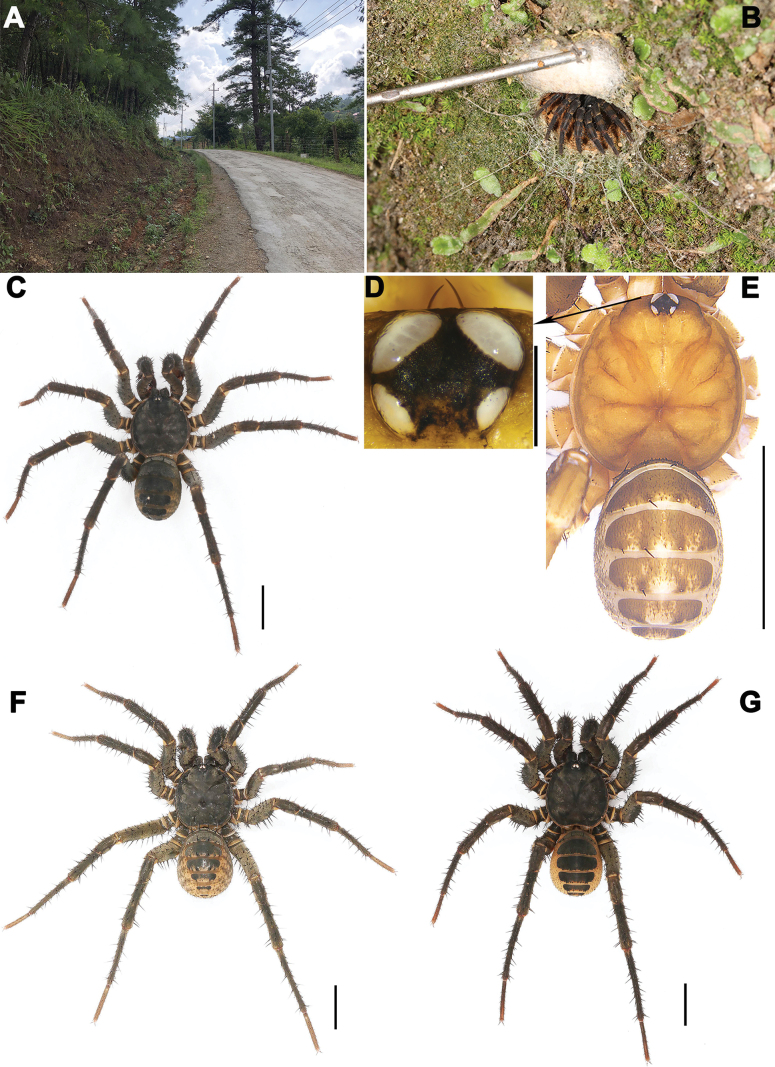
Microhabitat and general somatic morphology of four new *Liphistius* species. **A** microhabitat **B** burrow with trapdoor open **C, E–G** male dorsal view **C***L.kanpetlet* sp. nov. **D, E***L.rostratus* sp. nov. **F***L.kalaw* sp. nov. **G***L.nawngau* sp. nov. Scale bars: 0.5 mm (**D**); 5 mm (**C, E–G**).

Following an examination of specimens collected from Myanmar, we identify and describe four new *Liphistius* species, each restricted to a limited range, all belong to the *birmanicus*-group.

## ﻿Material and methods

All specimens were collected alive in Myanmar (Figs 1, 2) and transported the subadult individuals to the laboratory, where they were reared until reaching maturity. Right legs were removed from adults, preserved in absolute ethanol, and stored at −80 °C for genome DNA extraction. The remaining parts of each specimen were preserved in 80% ethanol as vouchers for morphological examination. These vouchers are currently deposited at the School of Life Sciences, Hubei University, Wuhan, Hubei Province, China (**HUBU**). In the future, the specimens will be deposited at two locations: School of Life Sciences, Hubei University, Wuhan, Hubei Province, China, and the Southeast Asia Biodiversity Research Institute, Chinese Academy of Sciences, Yezin, Nay Pyi Taw, Myanmar (CAS-SEABRI).

For morphological examination, we used an Olympus SZ51 stereomicroscope to dissect the specimens. Soft tissues of vulvae were removed using 10 mg/ml pancreatin, allowing for a minimum 3-hour digestion period at room temperature. Male palps and female genitalia were photographed using an Olympus BX53 compound microscope equipped with a digital camera CCD. Compound-focused images were generated using Helicon Focus v. 6.7.1. Specimen coloration was described in alcohol. All measurements were carried out under a Leica M205C stereomicroscope using Leica Application Suite v. 4 software and are given in millimeters. Measurements of legs and palp are presented in the following order: leg total length (femur, patella, tibia, metatarsus [absent on palp], tarsus).

Genital anatomical terminology used in this paper follows Aung et al. 2019 and Schwendinger et al. 2019, 2022. Abbreviations used in the text are: ALE = anterior lateral eyes; AME = anterior median eyes; PLE = posterior lateral eyes; PME = posterior median eyes; BL = body length (excluding chelicerae); CL = carapace length; OL = opisthosoma length; CW = carapace width; OW = opisthosoma width.

## ﻿Taxonomy

**Family Liphistiidae Thorell, 1869**

### ﻿Genus Liphistius

Taxon classificationAnimaliaAraneaeLiphistiidae

Schiödte, 1849

043B9D93-15DA-54B4-9D44-6F37D5218DCF

#### Type species.

*Liphistiusdesultor* Schiödte, 1849 from Malaysia.

#### Diagnosis.

*Liphistius* can be distinguished from the other seven genera by having the male palps bearing tibial apophysis (TiA) (Figs 3A, 5C, 7C, 9A), clavate trichobothria on the dorsal side of cymbia and leg tarsi, and vulvae possessing median receptacular cluster (RC) and sclerotized poreplate (PPl) (Figs 4, 6, 8, 10).

#### Distribution.

China (Yunnan Province), Indonesia (Sumatra), Laos, Myanmar, Peninsular Malaysia, and Thailand.

#### Comments on *Liphistius* in Myanmar.

Eleven named *Liphistius* species in Myanmar were grouped into three species-groups: the *birmanicus*-group, comprising nine valid species; the *bristowei*-group, consisting of one known species; and the *trang*-group, also containing one known species (Schwendinger 1990; Schwendinger et al. 2022; Sivayyapram et al. 2024). All named *Liphistius* species of the *birmanicus*-group were described based on both sexes, except for *L.hpruso* Aung, Xu, Lwin, Sang, Yu, Liu, Liu & Li, 2019, which is only known from females. In this study, four new *Liphistius* species belonging to the *birmanicus*-group are described, identified according to the morphology of their copulatory organs in both sexes.

#### Composition of the *birmanicus*-group:

*L.birmanicus*, *L.cupreus* Schwendinger & Huber, 2022, *L.ferox* Schwendinger & Huber, 2022, *L.hpruso*, *L.lahu* Schwendinger, 1998, *L.lordae* Platnick & Sedgwick, 1984, *L.metopiae* Schwendinger, 2022, *L.nabang* Yu, Zhang & Zhang, 2021, *L.pinlaung* Aung, Xu, Lwin, Sang, Yu, Liu, Liu & Li, 2019, *L.platnicki* Schwendinger & Huber, 2022, *L.pyinoolwin* Xu, Yu, Aung, Yu, Liu, Lwin, Sang & Li, 2021, *L.tung* Schwendinger, 2022.

### ﻿ Liphistius kalaw

Taxon classificationAnimaliaAraneaeLiphistiidae

Zhan & Xu sp. nov.

D0DEE7F1-43A9-512D-8994-2689CD66F8DD

https://zoobank.org/DE92B21F-6A12-44AA-97C8-18EE985DB623

Figs 3 , 4

#### Type material.

***Holotype***: Myanmar ♂, Shan State, 16 km W of Kalaw Township; 20.70°N, 96.52°E, alt. 944 m; 27.07.2019; D. Li et al. leg.; XUX-2019-061A. ***Paratypes***: 8♀♀, same data as for holotype; 27.07.2019 and 15.07.2018; XUX-2019-062/063/064/065/066/067A/068; XUX-2018-124.

#### Diagnosis.

**Figure 3.**
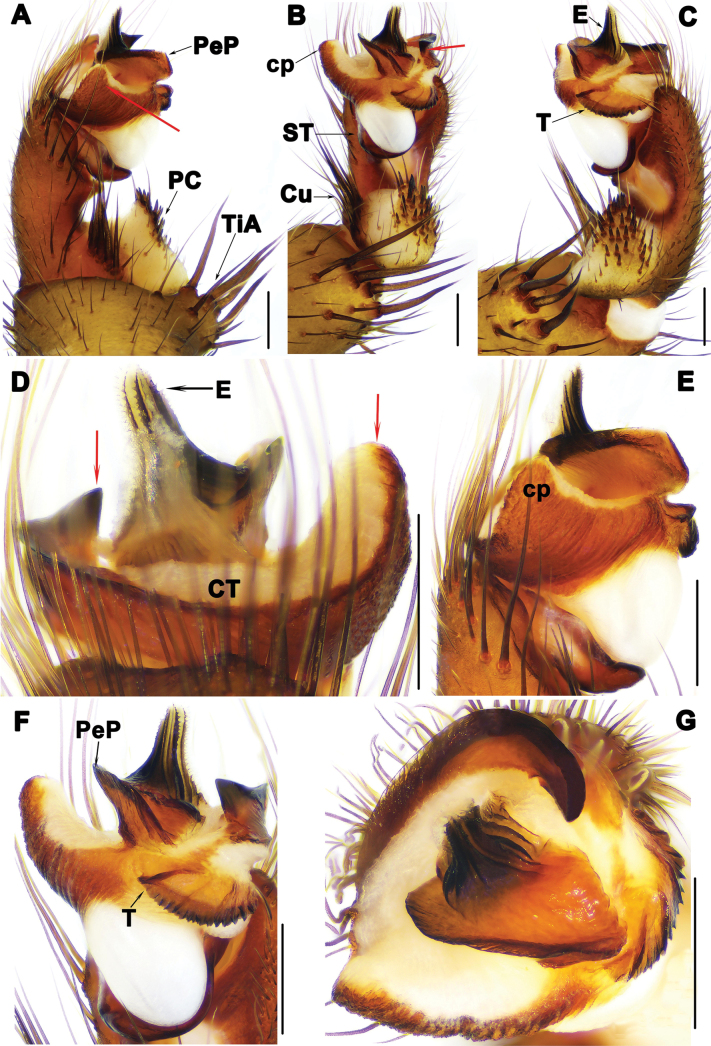
Male palp of *Liphistiuskalaw* sp. nov. **A, E** prolateral view **B** ventral view **C, F** retrolateral view **D** dorsal view, red arrows show arched projection and triangular process of CT**G** distal view. Abbreviations: CT = contrategulum; cp = contrategular process; Cu = cumulus; E = embolus; PC = paracymbium; PeP = paraembolic plate; ST = subtegulum; T = tegulum; TiA = tibial apophysis. Scale bars: 0.5 mm.

**Figure 4.**
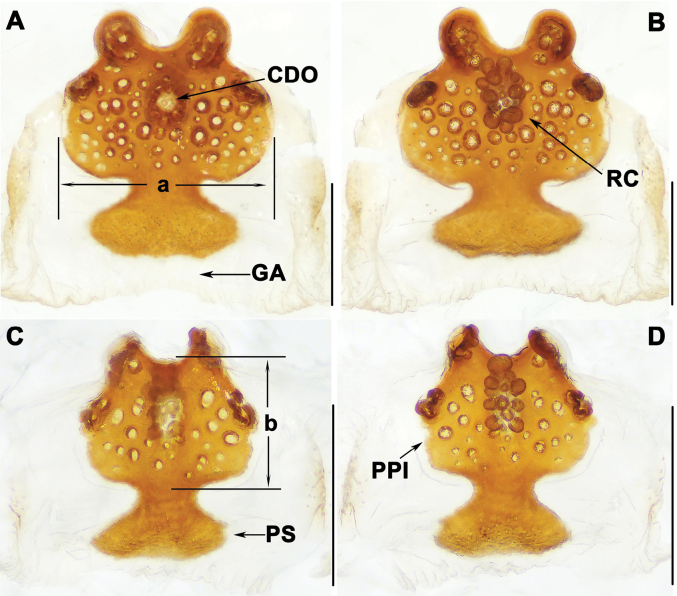
Vulva of *Liphistiuskalaw* sp. nov. **A, C** dorsal view **B, D** ventral view. Abbreviations: CDO = central dorsal opening; GA = genital atrium; PS = posterior stalk; PPl = poreplate; RC = receptacular cluster; a = length of poreplate transverse; b = width of poreplate. Scale bars: 0.5 mm.

The male of *L.kalaw* sp. nov. resembles those of *L.birmanicus* and *L.pinlaung* in having a distinct contrategular process (cp) (Fig. 3B, E), but it can be distinguished from *L.birmanicus* by the relatively wider base of the contrategular process (cp) (Fig. 3E vs fig. 13E in Schwendinger et al. 2022), and from *L.pinlaung* by the cumulus (Cu) which has shorter spines (Fig. 3A–C vs fig. 4C in Aung et al. 2019). The female of *L.kalaw* sp. nov. can be distinguished from that of *L.hpruso* by the relatively wider posterior stalk (PS) (Fig. 4C vs fig. 3B–E in Aung et al. 2019).

#### Description.

**Male holotype.** Carapace yellowish brown, with few short, scattered bristles; opisthosoma yellowish brown, with 12 brown tergites, close to each other, 2–6^th^ larger than others, 5^th^ largest; chelicerae robust, promargin with 13 denticles of variable size; labium and sternum yellowish brown, with few short setae on anterior margin and many long setae on elongated posterior margin; legs yellowish brown, with strong setae and spines. Measurements: BL 13.65, CL 5.94, CW 6.07, OL 8.00, OW 5.36; eye sizes and interdistances: AME 0.08, ALE 0.54, PME 0.27, PLE 0.43, AME–AME 0.11, AME–ALE 0.15, PME–PME 0.08, PME–PLE 0.10, ALE–PLE 0.17, ALE–ALE 0.12, PLE–PLE 0.34, AME–PME 0.10; labium 1.06 long and 0.98 wide; sternum 3.08 long and 0.96 wide; legs: I 18.36 (4.81, 2.57, 3.87, 4.60, 2.51), II 20.24 (5.64, 2.56, 4.23, 5.07, 2.74), III 20.46 (5.64, 2.37, 4.42, 6.00, 3.03), IV 27.56 (6.97, 2.84, 5.57, 8.17, 4.01).

Palp: tibial apophysis (TiA) with 4 tapering setae of similar length (Fig. 3C); paracymbium (PC) with many setae situated on tip (Fig. 3A–C); cumulus (Cu) slightly elevated, with 8 tapering spines (Fig. 3A–C); subtegulum with weakly developed apophysis (Fig. 3B); contrategulum (CT) with arched projection distally, and large triangular process in prolateral view (Fig. 3A, B, D); tegulum (T) fan-shaped with serrated edge and longitudinal ridges (Fig. 3C, F); paraembolic plate (PeP) narrow, sclerotized (Fig. 3A, F); embolus (E) with 4 distinct longitudinal ridges reaching tip in retrolateral view, and with several denticles along longitudinal ridges (Fig. 3B–F).

**Female paratype (XUX-2019-062).** Carapace yellowish brown, with few short, scattered bristles; opisthosoma brown, with 12 brown tergites, close to each other, with gray patches, 2–6^th^ larger than others, 5^th^ largest; chelicerae robust, reddish brown; promargin of cheliceral groove with 12 denticles of variable size; labium yellowish brown, sternum with several setae; legs with strong setae and spines. Measurements: BL 11.56, CL 5.82, CW 5.22, OL 6.23, OW 4.62; eye sizes and interdistances: AME 0.07, ALE 0.56, PME 0.29, PLE 0.42, AME–AME 0.09, AME–ALE 0.15, PME–PME 0.06, PME–PLE 0.09, ALE–PLE 0.11, ALE–ALE 0.07, PLE–PLE 0.35, AME–PME 0.08; labium 3.00 long and 0.61 wide; sternum 1.19 long and 1.13 wide; palp 10.25 (3.62, 1.74, 2.36, 2.53), legs: I 12.78 (4.29, 2.03, 2.63, 2.37, 1.46), II 12.89 (4.17, 2.03, 2.56, 2.59, 1.54), III 13.42 (4.13, 1.84, 2.75, 2.78, 1.92), IV 19.33 (5.57, 2.32, 3.81, 4.91, 2.72).

Vulva: poreplate (PPl) slightly wider than long, with pair of large anterior lobes and pair of small anterolateral lobes; central dorsal opening (CDO) circular, racemose receptacular cluster (RC) slightly long; posterior stalk (PS) axe-shaped; lateral margins of genital atrium (GA) membranous (Fig. 4).

#### Variation.

Females vary in body size. The range measurements of females (*N* = 8): BL 8.55–16.11, CL 4.48–7.20, CW 3.90–6.43, OL 4.18–8.56, OW 3.00–7.36. The number of denticles on the promargin of cheliceral groove varies from 11 to 14 (*N* = 8).

#### Etymology.

The species epithet “kalaw” refers to the type locality, Kalaw Township; it is treated as a noun in apposition.

#### Distribution.

Mandalay Region (Kalaw Township), Myanmar.

### ﻿ Liphistius kanpetlet

Taxon classificationAnimaliaAraneaeLiphistiidae

Zhan & Xu sp. nov.

A5B909E3-5575-5A20-8FD7-9858B83ECEA8

https://zoobank.org/8982C984-06DB-4636-9C22-92E2C69A62CB

Figs 5 , 6

#### Type material.

***Holotype***: Myanmar ♂, Chin State, Kanpetlet Township, Kanpetlet Matupi Rd; 21.19°N, 94.05°E, alt. 1469 m; 17.07.2019; D. Li et al. leg.; XUX-2019-026. ***Paratypes***: 5♀♀, same locality as for holotype; 17–18.07.2019; XUX-2019-020/021/024/031/033.

#### Diagnosis.

**Figure 5.**
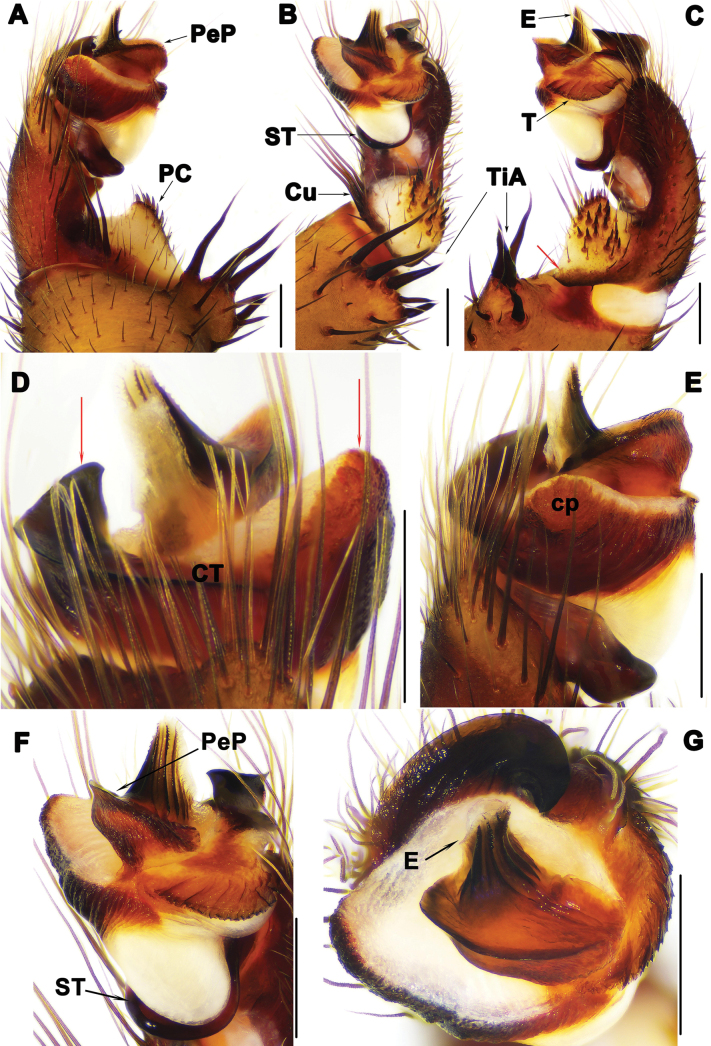
Male palp of *Liphistiuskanpetlet* sp. nov. **A, E** prolateral view **B** ventral view; **C, F** retrolateral view, red arrow shows distal process of PC**D** dorsal view, red arrows show arched projection and triangular process of CT**G** distal view. Abbreviations: CT = contrategulum; cp = contrategular process; Cu = cumulus; E = embolus; PC = paracymbium; PeP = paraembolic plate; ST = subtegulum; T = tegulum; TiA = tibial apophysis. Scale bars: 0.5 mm.

**Figure 6.**
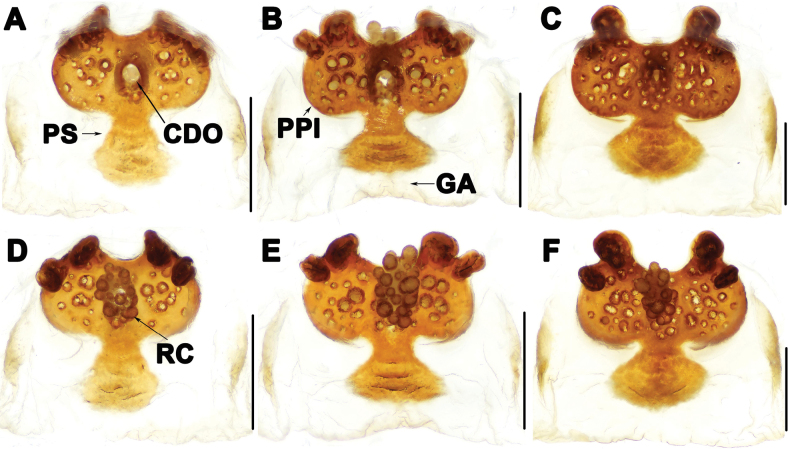
Vulva of *Liphistiuskanpetlet* sp. nov. **A–C** dorsal view **D–F** ventral view. Abbreviations: CDO = central dorsal opening; GA = genital atrium; PS = posterior stalk; PPl = poreplate; RC = receptacular cluster. Scale bars: 0.5 mm.

The male of *L.kanpetlet* sp. nov. can be distinguished from those of *L.birmanicus* by having the paracymbium (PC) bearing a distal process (Fig. 5C vs lacking a distal process, fig. 13H in Schwendinger et al. 2022), and from *L.kalaw* sp. nov. by the contrategulum (CT) which has a blunt process (Fig. 5D vs slightly sharp process, Fig. 3E). The female of *L.kanpetlet* sp. nov. differs from that of *L.hpruso* by having two relatively larger anterior lobes (Fig. 6 vs fig. 3B–E in Aung et al. 2019).

#### Description.

**Male holotype.** Carapace yellowish brown with few short, scattered bristles; opisthosoma brown, with 12 brown tergites, close to each other, 2–6^th^ larger than others, 4^th^ largest; chelicerae robust, promargin of cheliceral groove with 13 denticles of variable size; labium yellow, sternum yellow, with few short setae on anterior tip and many long setae on elongated posterior tip; legs reddish brown, with strong setae and spines; 8 spinnerets. Measurements: BL 12.41, CL 6.01, CW 6.06, OL 6.30, OW 4.19; eye sizes and interdistances: AME 0.06, ALE 0.56, PME 0.32, PLE 0.42, AME–AME 0.06, AME–ALE 0.16, PME–PME 0.10, PME–PLE 0.11, ALE–PLE 0.14, ALE–ALE 0.14, PLE–PLE 0.34, AME–PME 0.08; labium 0.96 long and 0.50 wide; sternum 2.63 long and 1.00 wide; legs: I 17.51 (4.82, 2.52, 3.79, 4.14, 2.24), II 18.36 (5.20, 2.19, 3.95, 4.72, 2.3), III 20.63 (5.46, 2.20, 4.41, 5.86, 2.70), IV 26.62 (7.18, 2.64, 5.40, 7.71, 3.69).

Palp: tibial apophysis (TiA) with 5 setae of different lengths (Fig. 5A–C); paracymbium (PC) with some setae situated on tip, and with pointed lateral process (Fig. 5C); and several tapering spines on slightly elevated cumulus (Cu) (Fig. 5B); subtegular apophysis weakly developed (Fig. 5B, F); contrategulum (CT) with arched projection distally, and wide triangular process in prolateral view (Fig. 5D); fan-shaped tegulum (T) with 6 longitudinal ridges (Fig. 5C); paraembolic plate (PeP) widely rounded, short, with curved margin in ventral view (Fig. 5A, F); embolus (E) sclerotized basally, with several denticles along longitudinal ridges reaching tip (Fig. 5C–G).

**Female paratype (XUX-2019-024).** Carapace brown with few short, scattered bristles; opisthosoma gray, with 12 brown tergites, close to each other, with gray patches, 2–6^th^ larger than others, 5^th^ largest; chelicerae robust, reddish brown; promargin of cheliceral groove with 10 denticles of variable size; labium yellow, sternum yellow with several setae; legs with strong setae and spines, without distinct annulations. Measurements: BL 11.71, CL 5.26, CW 5.15, OL 6.36, OW 4.70; eye sizes and interdistances: AME 0.09, ALE 0.50, PME 0.34, PLE 0.40, AME–AME 0.06, AME–ALE 0.19, PME–PME 0.07, PME–PLE 0.10, ALE–PLE 0.09, ALE–ALE 0.14, PLE–PLE 0.35, AME–PME 0.06; Labium 1.38 long and 0.59 wide; sternum 2.31 long and 1.19 wide; palp 8.05 (2.76, 1.36, 2.15, 1.78), leg I 10.24 (3.21, 1.60, 2.30, 2.01, 1.12), II 10.82 (3.43, 1.68, 2.27, 2.14, 1.30), III 11.14 (3.49, 1.51, 2.16, 2.51, 1.47), IV 15.54 (4.45, 1.94, 3.13, 3.71, 2.31).

Vulva: approximately rectangular poreplate (PPl) wider than long with smoothly curved posterior margin; with pair of large anterior lobes and pair of small anterolateral lobes, 2 anterior lobes separated from each other, but close to anterolateral lobes; central dorsal opening (CDO) small, situated in center of poreplate (PPl); racemose receptacular cluster (RC) long and narrow; posterior stalk (PS) axe-shaped; genital atrium (GA) with slightly sclerotized lateral margins (Fig. 6).

#### Variation.

Females vary in body size. The range of measurements of females (*N* = 5): BL 9.70–14.85, CL 4.86–6.55, CW 4.20–8.63, OL 3.82–8.63, OW 3.82–7.09. The number of denticles on the promargin of cheliceral groove varies from 10–13 (*N* = 5).

#### Etymology.

The species epithet “kanpetlet” refers to the type locality, Kanpetlet Township; it is treated as a noun in apposition.

#### Distribution.

Chin State (Kanpetlet Township), Myanmar.

### ﻿ Liphistius nawngau

Taxon classificationAnimaliaAraneaeLiphistiidae

Zhan & Xu sp. nov.

1148FBC4-05EA-553C-96A0-DBAFAAE5E4F3

https://zoobank.org/1ADFCE8F-66EE-43F9-B61A-927F6B88189F

Figs 7 , 8

#### Type material.

***Holotype***: Myanmar ♂, Shan State, Kyaukme Dist., Nawnghkio Township, Nawng Au Vill.; 22.26°N, 96.83°E, alt. 1096 m; 26.07.2019; D. Li et al. leg.; XUX-2019-054A. ***Paratypes***: 9♂♂ 3♀♀, same data as for holotype; XUX-2019-054/054A/055/055A/055B/055D/056/056A/057/058/059/060; 5♂♂ 2♀♀, same township as for holotype; 22.30°N, 96.73°E, alt. 845 m; 26.07.2019 and 14.07.2018; XUX-2019-049/051/052/053, XUX-2018-116/119/123.

#### Diagnosis.

**Figure 7.**
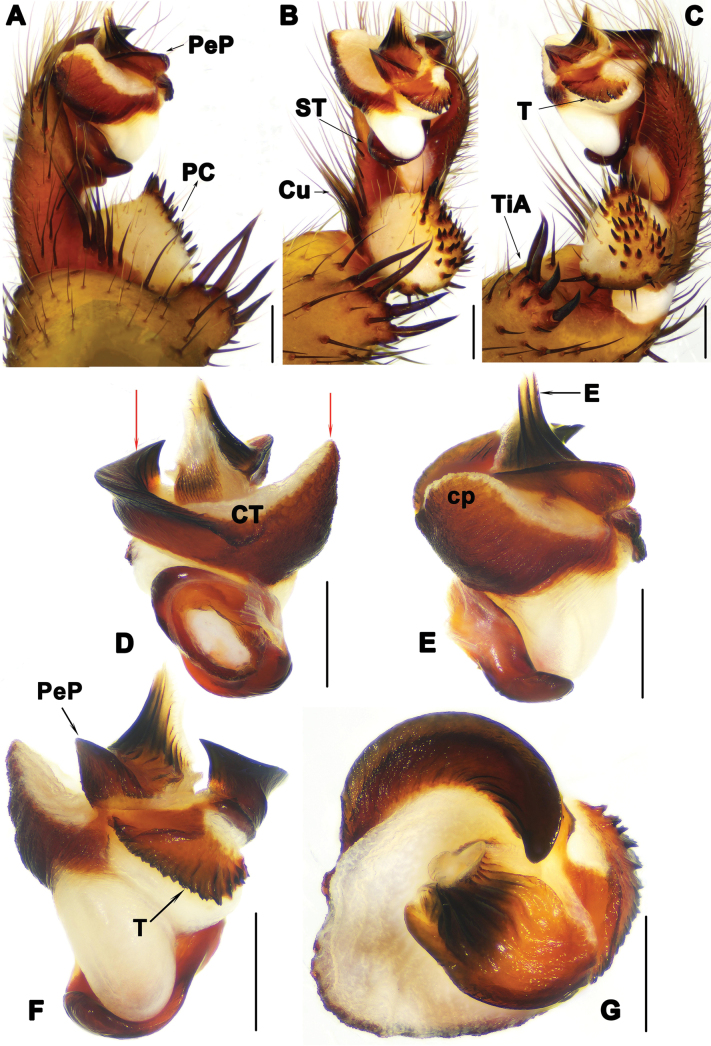
Male palp of *Liphistiusnawngau* sp. nov. **A, E** prolateral view **B** ventral view **C, F** retrolateral view **D** dorsal view, red arrows show arched projection and triangular process of CT**G** distal view. Abbreviations: CT = contrategulum; cp = contrategular process; Cu = cumulus; E = embolus; PC = paracymbium; PeP = paraembolic plate; ST = subtegulum; T = tegulum; TiA = tibial apophysis. Scale bars: 0.5 mm.

**Figure 8.**
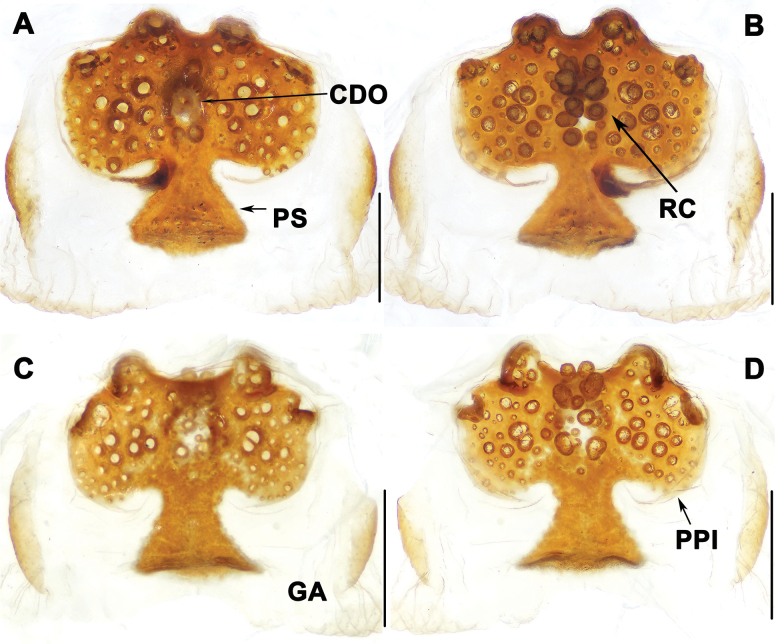
Vulva of *Liphistiusnawngau* sp. nov. **A, C** dorsal view **B, D** ventral view. Abbreviations: CDO = central dorsal opening; GA = genital atrium; PS = posterior stalk; PPl = poreplate; RC = receptacular cluster. Scale bars: 0.5 mm.

The male of *L.nawngau* sp. nov. resembles those of *L.lordae* and *L.pyinoolwin* in having an adpressed proximal tegular margin (Fig. 7C, F), but it can be distinguished from them by the tegulum (T), which bears a distinct transversal ridge in retrolateral view (Fig. 7C, F vs lacking transversal ridge, figs 5, 6 in Schwendinger 1990; fig. 4F in Xu et al. 2021), and by the embolus (E), which has smooth longitudinal ridges reaching tip (Fig. 7E vs having several denticles along longitudinal ridges, fig. 4F in Xu et al. 2021). The female of *L.nawngau* sp. nov. can be distinguished from that of *L.hpruso* by having the posterior stalk triangular (PS) (Fig. 8 vs nearly oval, fig. 3B–E in Aung et al. 2019).

#### Description.

**Male holotype.** Carapace brown with few short, scattered bristles; opisthosoma brown, with 12 brown tergites, close to each other, 2–6^th^ larger than others, 5^th^ largest; chelicerae robust, promargin of cheliceral groove with 11 denticles of variable size; labium and sternum yellowish brown, sternum with few short setae on anterior tip and many long setae on elongated posterior tip; legs yellowish brown, with strong setae and spines. Measurements: BL 15.47, CL 6.63, CW 6.57, OL 7.73, OW 5.56; eye sizes and interdistances: AME 0.08, ALE 0.72, PME 0.35, PLE 0.49, AME–AME 0.08, AME–ALE 0.20, PME–PME 0.08, PME–PLE 0.07, ALE–PLE 0.81, ALE–ALE 0.11, PLE–PLE 0.16, AME–PME 0.12; labium 0.81 long and 0.58 wide; sternum 2.59 long and 1.01 wide; legs: I 19.58 (5.67, 2.78, 4.03, 4.68, 2.42), II 20.42 (5.66, 2.78, 4.13, 4.96, 2.89), III 22.62 (5.96, 2.63, 4.54, 6.29, 3.20), IV 28.26 (7.16, 2.75, 5.82, 8.55, 3.98).

Palp: tibial apophysis (TiA) with four setae of similar length (Fig. 7A–C); paracymbium (PC) with several setae situated on tip (Fig. 7A–C); cumulus (Cu) slightly elevated, with 8 tapering spines (Fig. 7A–C); subtegulum (ST) with weakly developed apophysis (Fig. 7B, F); contrategulum (CT) with an arched projection distally, and triangular process with wide base (Fig. 7D); tegulum (T) with distinct transversal ridge in retrolateral view (Fig. 7C, F); paraembolic plate (PeP) narrow, sclerotized (Fig. 7A, F); embolus (E) with several longitudinal ridges reaching tip (Fig. 7E, F).

**Female paratype (XUX-2019-053).** Carapace yellowish brown with few short, scattered bristles; opisthosoma brown, with 12 brown tergites, close to each other, with gray patches, 2–6^th^ larger than others, 5^th^ largest; chelicerae robust, reddish brown; promargin of cheliceral groove with 12 denticles of variable size; labium and sternum yellowish brown; legs with strong setae and spines. Measurements: BL 14.24, CL 6.10, CW 5.30, OL 7.12, OW 4.96; eye sizes and interdistances: AME 0.08, ALE 0.57, PME 0.31, PLE 0.45, AME–AME 0.10, AME–ALE 0.17, PME–PME 0.09, PME–PLE 0.08, ALE–PLE 0.09, ALE–ALE 0.10, PLE–PLE 0.46, AME–PME 0.06; labium 1.23 long and 0.77 wide; sternum 2.68 long and 1.13 wide; palp 10.42 (3.63, 1.84, 2.77, 2.18), leg I 13.33 (4.16, 2.15, 2.90, 2.58, 1.54), II 16.85 (4.35, 2.26, 2.84, 2.84, 1.72), III 15.04 (4.37, 2.35, 2.90, 3.31, 2.11), IV 20.39 (5.92, 1.92, 4.48, 5.33, 2.74).

Vulva: poreplate (PPl) with pair of large anterior lobes and pair of relatively small anterolateral lobes; central dorsal opening (CDO) located at center of poreplate (PPl); receptacular cluster (RC) racemose, protrudes upper edge of poreplate (PPl); posterior margin of triangular posterior stalk (PS) almost straight; lateral margins of genital atrium (GA) slightly sclerotized (Fig. 8).

#### Variation.

Males (*N* = 14): BL 12.64–16.98, CL 6.27–7.56, CW 6.02–7.56, OL 6.95–8.50, OW 4.60–6.40; females (*N* = 5): BL 10.55–14.24, CL 5.26–6.37, CW 4.47–5.63, OL 5.24–7.12, OW 4.33–4.96. The number of denticles on the promargin of male cheliceral groove varies from 10 to 13 (*N* = 12); in females, the number of denticles on the promargin of cheliceral groove varies from 11 to 12 (*N* = 5). The number of setae on tibial apophysis varies from 4 to 6.

#### Etymology.

The species epithet “nawngau” refers to the type locality, Nawng Au Village; it is treated as a noun in apposition.

#### Distribution.

Shan State, (Kyaukme District), Myanmar.

### ﻿ Liphistius rostratus

Taxon classificationAnimaliaAraneaeLiphistiidae

Zhan & Xu sp. nov.

BE9AF2F0-5BBA-5A87-B548-EEDB4256870E

https://zoobank.org/35E36D61-84CB-4C01-830F-BCFE53FBAA6E

Figs 9 , 10

#### Type material.

***Holotype***: Myanmar ♂, Mandalay Region, War Phyu Taung Vill.; 22.88°N, 96.12°E, alt. 553 m; 25.07.2019; D. Li et al. leg.; XUX-2019-038. ***Paratypes***: 2♂♂ 3♀♀, same data as for holotype; XUX-2019-034–037/041.

#### Diagnosis.

**Figure 9.**
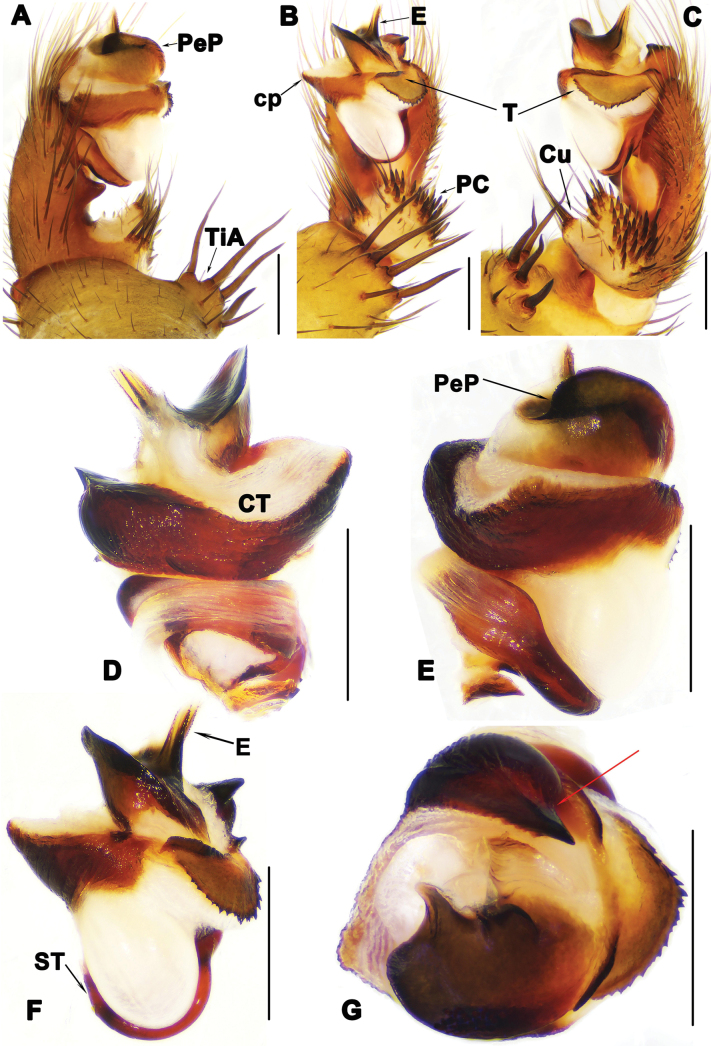
Male palp of *Liphistiusrostratus* sp. nov. **A, E** prolateral view **B** ventral view **C, F** retrolateral view **D** dorsal view **G** distal view, red arrow shows beak-like contrategular projection. Abbreviations: CT = contrategulum; cp = contrategular process; Cu = cumulus; E = embolus; PC = paracymbium; PeP = paraembolic plate; ST = subtegulum; T = tegulum; TiA = tibial apophysis. Scale bars: 0.5 mm.

**Figure 10.**
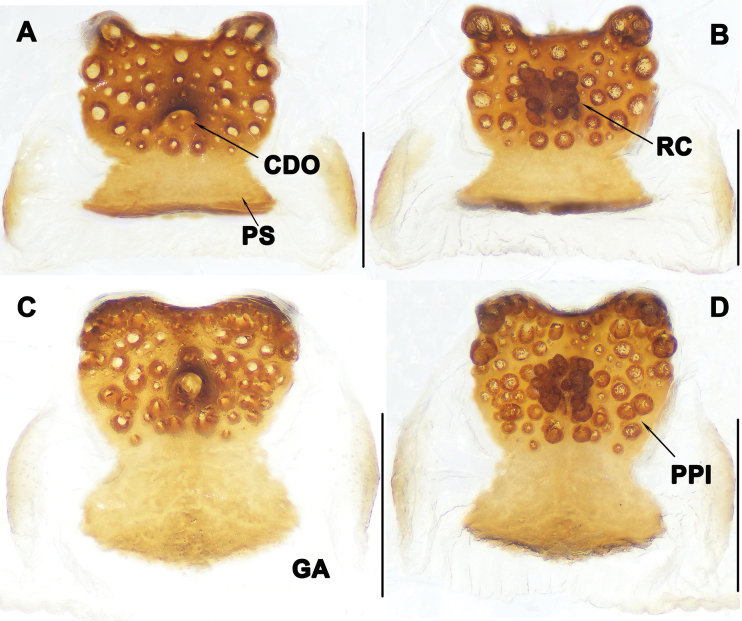
Vulva of *Liphistiusrostratus* sp. nov. **A, C** dorsal view **B, D** ventral view. Abbreviations: CDO = central dorsal opening; GA = genital atrium; PS = posterior stalk; PPl = poreplate; RC = receptacular cluster. Scale bars: 0.5 mm.

The male of *L.rostratus* sp. nov. resembles those of *L.cupreus*, *L.nabang*, and *L.platnicki* in having distinctly elevated cumulus (Cu) (Fig. 9C), but it can be distinguished from them by the relatively larger paraembolic plate (PeP) (Fig. 9A, E vs figs 21A, 23K in Schwendinger et al. 2022; fig. 3A in Yu et al. 2021), and an indistinct contrategular process (cp) (Fig. 9B vs relatively large contrategular process, figs 21E, 23E in Schwendinger et al. 2022; fig. 3B in Yu et al. 2021). The female of *L.rostratus* sp. nov. resembles to that of *L.cupreus* in having vesicle clusters along the anterior margin of the poreplate (PPl), but it can be distinguished by the relatively longer posterior stalk (PS), with a length/width ratio 0.4–0.6 (Fig. 10 vs length/width ratio about 0.2–0.4, fig. 22 in Schwendinger et al. 2022).

#### Description.

**Male holotype.** Carapace yellowish brown, with few short, scattered bristles; opisthosoma brown, with 12 brown tergites, close to each other, 2–6^th^ larger than others, 4^th^ largest; chelicerae robust, promargin of cheliceral groove with 12 denticles of variable size; labium and sternum yellowish brown, sternum with few short setae on anterior tip and many long setae on elongated posterior tip; legs yellowish brown, with strong setae and spines. Measurements: BL 9.34, CL 4.57, CW 4.26, OL 5.02, OW 3.59; eye sizes and interdistances: AME reduced, ALE 0.40, PME reduced, PLE 0.24, ALE–PLE 0.07, ALE–ALE 0.05, PLE–PLE 0.30; labium 0.98 long and 0.48 wide; sternum 2.09 long and 0.83 wide; legs: I 12.63 (3.80, 1.32, 2.60, 3.07, 1.84), II 13.19 (3.80, 1.32, 2.60, 3.07, 1.84), III 14.35 (3.79, 1.58, 3.33, 3.44, 2.21), IV 19.76 (4.76, 1.66, 4.13, 6.01, 3.20).

Palp: tibial apophysis (TiA) with 4 setae of similar length (Fig. 9A–C); paracymbium (PC) with several setae situated on tip (Fig. 9B, C); cumulus (Cu) distinct elevated with 5 tapering spines (Fig. 9C); subtegulum (ST) without apophysis (Fig. 9F); contrategulum (CT) with beak-like projection distally in distal view and small process in ventral view (Fig. 9B, G); tegulum (T) semicircular with flat surface and serrated edges, and proximal tegular margin with an elevated transverse ridge (Fig. 9B, C, F); paraembolic plate (PeP) sclerotized, narrow (Fig. 9A, E); embolus (E) with 3 distinct longitudinal ridges reaching tip retrolaterally, and several denticles along these longitudinal ridges (Fig. 9B, F).

**Female paratype (XUX-2019-034).** Carapace brown with few short, scattered bristles; opisthosoma brown, with 12 brown tergites, close to each other, 2–6^th^ larger than others, 5^th^ largest; chelicerae robust, reddish brown; promargin of cheliceral groove with 12 denticles of variable size; labium yellowish brown, sternum yellowish brown with several setae; legs with strong setae and spines. Measurements: BL 10.54, CL 4.5, CW 3.93, OL 6.47, OW 4.74; eye sizes and interdistances: AME 0.05, ALE 0.37, PME 0.19, PLE 0.29, AME–AME 0.07, AME–ALE 0.12, PME–PME 0.06, PME–PLE 0.06, ALE–PLE 0.09, ALE–ALE 0.10, PLE–PLE 0.32, AME–PME 0.08; labium 0.95 long and 0.38 wide; sternum 2.41 long and 0.99 wide; palp 7.10 (2.44, 0.98, 1.86, 1.82), leg I 8.89 (2.94, 1.34, 2.00, 1.62, 0.99), II 9.69 (3.40, 1.09, 2.02, 1.98, 1.20), III 9.48 (2.29, 1.05, 2.59, 2.38, 1.17), IV 13.71 (3.21, 1.44, 3.11, 3.80, 2.21).

Vulva: about rectangular poreplate (PPl) with pair of anterior lobes; central dorsal opening (CDO) located below center of poreplate (PPl); racemose receptacular cluster (RC) small; posterior stalk (PS) as wide as poreplate (PPl), lateral margins of genital atrium (GA) slightly sclerotized (Fig. 10).

#### Variation.

Males (*N* = 3): BL 9.34–10.31, CL 4.57–5.24, CW 4.26–5.83, OL 4.73–5.10, OW 3.40–3.40; females (*N* = 4): BL 10.54–12.35, CL 4.50–6.31, CW 3.93–5.47, OL 6.47–6.87, OW 4.64–4.79. The number of denticles on the cheliceral promargin varies from 12 to13 (*N* = 6). In addition, the male lacking an AME and PME, which may be degenerate during molting, is chosen as the holotype, because all paratype male palps are deformed.

#### Etymology.

The species name is derived from the Latin word “*rostratus*”, referring to the beak-like contrategulum of the male palp in distal view.

#### Distribution.

Mandalay Region (War Hpyu Taung), Myanmar.

## Supplementary Material

XML Treatment for Liphistius

XML Treatment for Liphistius kalaw

XML Treatment for Liphistius kanpetlet

XML Treatment for Liphistius nawngau

XML Treatment for Liphistius rostratus

## ﻿Additional information

### Author ORCIDs

Xin Xu https://orcid.org/0000-0001-5632-6622

Long Yu https://orcid.org/0000-0001-5675-1864

Daiqin Li https://orcid.org/0000-0001-8269-7734

### Data availability

All of the data that support the findings of this study are available in the main text.
